# 2777. Species Distribution and Antimicrobial Susceptibility of *Enterobacter* in Bacteremia of Korean Children, 2011-2022

**DOI:** 10.1093/ofid/ofad500.2388

**Published:** 2023-11-27

**Authors:** Da Yun Kang, Ye Eun Kim, Sung Hwan Choi, Eun Hwa Choi, Ki Wook Yun

**Affiliations:** Department of Pediatrics, Seoul National University Children’s Hospital, Seoul, South Korea, seoul, Seoul-t'ukpyolsi, Republic of Korea; Seoul National University Children's Hospital, Seoul, Seoul-t'ukpyolsi, Republic of Korea; Seoul National University Children's Hospital, Seoul, Seoul-t'ukpyolsi, Republic of Korea; Seoul National University Children's Hospital, Seoul, Seoul-t'ukpyolsi, Republic of Korea; Seoul National University Children's Hospital, Seoul, Seoul-t'ukpyolsi, Republic of Korea

## Abstract

**Background:**

*Enterobacter* is an increasingly important nosocomial pathogen due to the resistance to penicillin and cephalosporin mediated by a chromosomal (*ampC*) beta-lactamase. We aimed to identify the species distribution and antimicrobial susceptibility isolated from the blood of Korean children.

**Methods:**

Demographic, clinical, and laboratory data were retrospectively reviewed from patients under 21 hospitalized for bacteremia at Seoul National University Children's Hospital during 2011-2022. A VITEK II automated was used for species identification and antimicrobial susceptibility testing, and the susceptibility was determined according to the Clinical and Laboratory Standards Institute guidelines.

**Results:**

Bacteremia due to *Enterobacter* species was identified in 106 cases in a total of 102 patients. The proportion of patient’s age group was 33.0% in infants (< 1 year old), 17.9% in toddlers (1-4 years old), 22.6% in children (5-11 years old), and 26.4% in adolescents (12-20 years old). Species were 70.8% of *Enterobacter cloacae*, 23.6% of *K. aerogenes*, 3.8% of *Enterobacter asburiae*, and each one (0.9%) of *Enterobacter amnigenus* and *Enterobacter intermedius*. Overall, non-susceptibility to piperacillin-tazobactam (PIP-TAZ), cefotaxime (CTX), cefepime, and carbapenem were 27.4%, 36.8%, 7.5%, and 4.7%, respectively. The most susceptible antibiotic was amikacin (99.1%). Multidrug resistance (MDR) was identified in 16.0% of isolates. Although statistically non-significant, the non-susceptibilities to PIP-TAZ and CTX increased from 16.7% and 8.3% in 2013 to 60.0% and 40.0%, respectively, in 2022. In addition, the proportion of *E. cloacae* has significantly increased (*p* for trend=0.034) from 66.7% in 2014 to 91.7% in 2020. *K. aerogenes* is significantly (*p*=0.002) more common in infants than *E. cloacae*, and *E. cloacae* (17.3%) showed more than twice as many MDR feature as *K. aerogenes* (8.0%), although not statistically significant.

**Conclusion:**

Although *Enterobacter* species has been steadily detected as a cause of pediatric bacteremia, the rate of *E. cloacae* has been increasing recently. Considering antibiotic susceptibility data, cefepime alone or combination with a third-generation cephalosporin and amikacin might be reasonable empirical antibiotic therapy regimen.

**Disclosures:**

**All Authors**: No reported disclosures

